# Amorphous solid dispersions of enzalutamide and novel polysaccharide derivatives: investigation of relationships between polymer structure and performance

**DOI:** 10.1038/s41598-020-75077-7

**Published:** 2020-10-28

**Authors:** Venecia R. Wilson, Xiaochun Lou, Donald J. Osterling, DeAnne F. Stolarik, Gary J. Jenkins, Brittany L. B. Nichols, Yifan Dong, Kevin J. Edgar, Geoff G. Z. Zhang, Lynne S. Taylor

**Affiliations:** 1 Department of Industrial and Physical Pharmacy, College of Pharmacy, Purdue University, 575 Stadium Mall Drive, Lafayette, IN 47907 USA; 2 Drug Product Development, Research & Development, AbbVie, 1 N. Waukegan Road, North Chicago, IL 60064 USA; 3 Drug Metabolism and Pharmacokinetics, Research & Development, AbbVie, 1 N. Waukegan Road, North Chicago, IL 60064 USA; 4 Department of Chemistry, College of Science, Virginia Tech, 240 Kelly Hall, Blacksburg, VA 24061 USA; 5 Department of Sustainable Biomaterials, Virginia Tech, 230A Cheatham Hall, Blacksburg, VA 24061 USA

**Keywords:** Polymers, Drug development

## Abstract

Amorphous solid dispersion (ASD) is a widely employed formulation technique for drugs with poor aqueous solubility. Polymers are integral components of ASDs, but mechanisms by which polymers lead to the generation and maintenance of supersaturated solutions, which enhance oral absorption in vivo, are poorly understood. Herein, a diverse group of newly synthesized cellulose derivatives was evaluated for their ability to inhibit crystallization of enzalutamide, a poorly soluble compound used to treat prostate cancer. ASDs were prepared from selected polymers, specifically a somewhat hydrophobic polymer that was extremely effective at inhibiting drug crystallization, and a less effective, but more hydrophilic, crystallization inhibitor, that might afford better release. Drug membrane transport rate was evaluated in vitro and compared to in vivo performance, following oral dosing in rats. Good correlation was noted between the in vitro diffusion cell studies and the in vivo data. The ASD formulated with the less effective crystallization inhibitor outperformed the ASD prepared with the highly effective crystallization inhibitor in terms of the amount and rate of drug absorbed in vivo. This study provides valuable insight into key factors impacting oral absorption from enabling ASD formulations, and how best to evaluate such formulations using in vitro approaches.

## Introduction

The proportion of drugs designated as Biopharmaceutics Classification System (BCS) class II and IV compounds has increased in recent years; these poorly water soluble compounds now comprise a majority of the drugs in development^1,2^. Since a drug in an oral dosage form must first dissolve prior to absorption across the gastrointestinal epithelium, it is critical that formulation techniques are employed to enhance dissolution rate and/or solubility; supersaturating formulations are of increasing interest for this purpose. A supersaturated solution occurs when the solute concentration exceeds the equilibrium solubility of the stable crystalline form. The increased concentrations achieved through generation of a supersaturated solution, in turn, improve the oral absorption of the drug. However, a major disadvantage of a supersaturated solution in terms of enhancing drug delivery, is its metastability and inherent tendency for the drug to crystallize, leading to a loss in solubility advantage. Amongst supersaturating formulation strategies, amorphous solid dispersions comprising a molecular level blend of drug and polymer have demonstrated improved bioavailability in vivo as compared to crystalline systems^3–6^. While the exact mechanisms by which drug dissolution from an ASD leads to supersaturated solutions are not fully understood, it is generally recognized that the polymer’s role is to facilitate drug release from the amorphous matrix and to delay subsequent crystallization^7,8^. The latter aspect is particularly important for rapidly crystallizing drugs because once crystallization commences, supersaturation is depleted and any solubility advantage is lost. Thus, it is generally considered important that the polymer contains both hydrophobic substituent groups to drive interaction with the drug in an aqueous environment, preventing crystallization, and hydrophilic groups to interact with water and facilitate drug release from the ASD^9–11^.

There is currently a limited number of polymers that have been used in commercial ASD dosage forms; the majority of Food and Drug Administration (FDA) approved ASDs are formulated with hydroxypropyl methyl cellulose (hypromellose, or HPMC), hydroxypropyl methyl cellulose acetate succinate (hypromellose acetate succinate, or HPMCAS), or poly(vinyl pyrrolidinone-*co*-vinyl acetate) (copovidone, or PVPVA)^12–14^. However, this small group of polymers is not sufficiently structurally diverse to enable systematic study of structure activity relationships, and these polymers were not specifically designed for ASD formulation, but rather were repurposed from other pharmaceutical applications. In recent years, synthesis of novel polymers specifically designed for use in ASDs, as well as to facilitate mechanistic understanding of key polymer functionality, has led to an increase in polymer diversity^11,15^. Ultimately, if polymers with enhanced properties can be identified, this may permit a higher proportion of poorly soluble drug candidates to be successfully formulated as ASDs. Further, if improved mechanistic understanding of drug release and crystallization inhibition can be realized, in the future polymers could be rationally designed and selected based upon the physiochemical and structural properties of the drug. One challenge worth noting is the difficulty in achieving adequate, sufficiently rapid drug release from a high drug loading ASD^16^. A number of causes of this difficulty can be imagined; a higher proportion of drug will alter the hydrophobicity, thermal properties, and water permeation rates into the dispersion, among other pertinent properties. The resulting increase in drug concentration will increase the risk of crystallization, while the concomitant decrease in polymer concentration means that proportionately less polymer is available to stabilize the drug against crystallization. However, achieving a high drug loading is desirable from a patient compliance perspective; low drug loading formulations contain a large amount of polymer, which increases dosage size and makes it difficult for the patient to swallow the oral dosage form.

In order to inhibit crystallization, the polymer must interact with the drug through specific interactions such as van der Waals forces, ionic interactions, and hydrogen bonding^9, 17,18^. It is to be expected that these types of interactions may differ in relative importance in the dry state versus the hydrated system. The polymer must dissolve to a concentration sufficient to provide interaction with drug and prevent recrystallization, but it has also been found that high polymer solubility, with subsequent rapid drug release, may lead to fast drug crystallization^19^ since highly water soluble polymers may show less tendency to interact with hydrophobic drugs^20^. At the same time, poorly water soluble polymers are unsuitable as they may limit the amount of drug released, leading to inadequate levels of supersaturation^21^. Given these opposing, critical performance criteria, it is therefore unsurprising that it is a complex problem to design polymers with an appropriate balance of functional groups to achieve the desired ASD performance in terms of both drug release and crystallization inhibition. In addition, the required balance between these two factors is currently uncertain. Moreover, it is unclear which in vitro tests accurately predict in vivo performance, with recent studies suggesting that membrane transport rate (flux) measurements may provide greater insight than simple dissolution tests^6,22,23^.

While the Taylor and Edgar groups have described design and in vitro testing of a number of new polysaccharide derivatives for ASDs of poorly soluble drugs^9,11,15,24,25^, no in vivo studies have been performed on formulations containing these polymers. The goal of this study was to evaluate in vivo absorption performance of two of these new polymers, selected from a larger group which were first evaluated in terms of their ability to inhibit crystallization during in vitro studies. Of particular interest, was the in vivo performance following oral dosage of formulations containing a high drug loading. Enzalutamide, a BCS class II compound used to treat prostate cancer, was selected as the model compound for the amorphous solid dispersion formulations with the new polymers. Enzalutamide is a lipophilic compound and does not ionize over physiologically relevant pH conditions. The commercial formulation of this compound is a lipid-based formulation in a soft gel capsule. The drug loading is low and hence patients have a high “pill burden” whereby they have to take four large capsules (capsule size is 9 mm × 20 mm). Therefore, increasing the drug loading without compromising the amount of drug absorbed is of considerable interest. Herein, structurally diverse polymers including many designed by us for ASD were first studied for their ability to inhibit crystallization of enzalutamide from supersaturated solutions by measuring the nucleation induction time. From these results, two newly synthesized cellulose derivatives with a different balance of hydrophobic and hydrophilic moieties were selected for ASD formulation. Relative polymer hydrophilicity was evaluated by measuring aqueous solubility and comparing solubility parameters. Permeation ability of the drug from the formulations was measured using a side-by-side diffusion cell to measure flux which reflects the extent of supersaturation achieved. The amount of drug absorbed in vivo was determined by dosing the different formulations to rats and determining plasma drug levels. A lipid formulation, similar to the commercial formulation, was also dosed.

## Results

### Nucleation induction times

**Figure 1:**
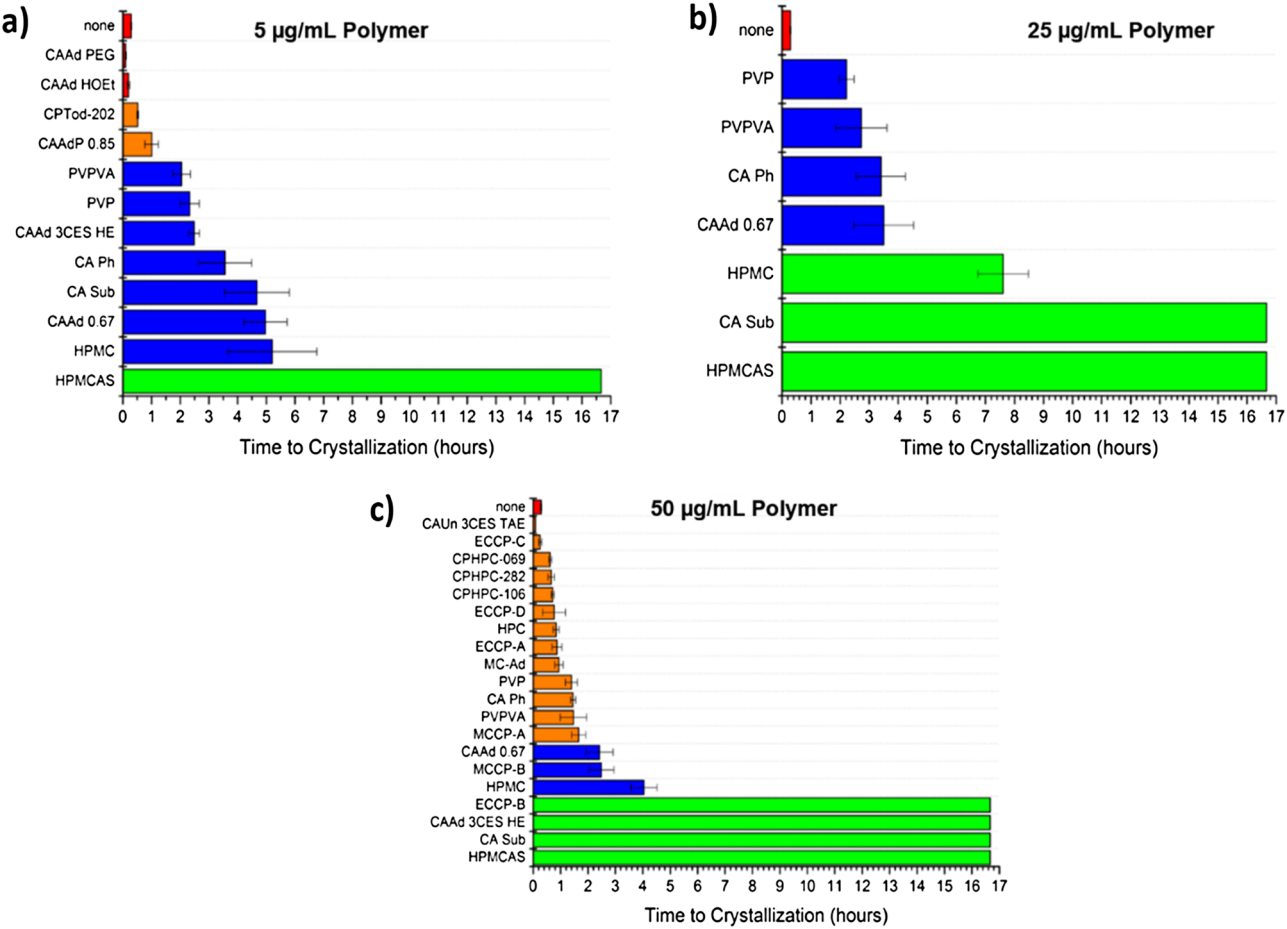
Average time to crystallization for supersaturated enzalutamide solutions (initial concentration 70 μg/mL) in presence of (**a**) 5, (**b**) 25, and (**c**) 50 μg/mL of pre-dissolved polymer in buffer. Polymers shown in red had induction times similar to that of enzalutamide alone (0–30 min), those in orange 30 min–2 h, blue 2–6 h, and green > 6 h.

Induction times of enzalutamide at an initial concentration corresponding to approximately 1.5× the amorphous solubility were determined in the presence of 3 different polymer concentrations: 5 μg/mL, 25 μg/mL, and 50 μg/mL. Results are summarized in Fig. 1. When 70 μg/mL of enzalutamide is added to the buffer solution, approximately 42 μg/mL enzalutamide exists as free drug molecularly dissolved, while the remaining enzalutamide is present as colloidal amorphous aggregates^6^. Consequently, to be optimally effective, the polymer must both inhibit crystallization of free drug in solution, and inhibit agglomeration or crystallization from the amorphous aggregates. Enzalutamide crystalline solubility is 2.9 μg/mL, and the supersaturation ratio (S, the ratio of amorphous solubility/crystalline solubility) is approximately 14.5. In the absence of polymers, the drug crystallized rapidly, with an induction time of ~ 15 min. HPMCAS inhibited crystallization for at least 16 h, irrespective of polymer concentration. Three of the novel cellulose derivatives, CAAd 3CES HE, CA Sub, and ECCP-B, also inhibited crystallization for > 16 h at the highest polymer concentration tested (50 μg/mL). Several polymers inhibited crystallization for > 2 h, and can therefore theoretically have a large impact on supersaturation duration in vivo where small intestine transit times has been reported to be approximately 2–4 h^26,27^. Crystallization inhibition effectiveness depended on polymer concentration for some of the polymers, in particular CA Sub; this polymer delayed crystallization for approximately 5 h at 5 μg/mL and inhibited crystallization for > 16 h at higher concentrations. In contrast, the effectiveness of several other polymers as crystallization inhibitors (CAAd 0.67, CA Ph, PVPVA, PVP, HPMCAS and HPMC) did not show dependence on polymer concentration.

The following structural features are common among the four polymers which most effectively inhibited crystallization at a concentration of 50 μg/mL: (1) cellulose backbone, (2) carbon chain in tether contains branched structures, (3) carboxylate group is located at the terminal end(s) of the tether, and (4) number of carbons in the tether is 6 or less. While these features were common to the most effective crystallization inhibitors, their inclusion does not guarantee that the polymer is effective as seen from evaluation of the structures of MC-Ad, MCCP polymers, and three of the ECCP polymers. Those polymers had approximately the same effectiveness at delaying enzalutamide crystallization as the non-cellulose derivatives, PVP and PVPVA (which has been widely used in ASD formulations)^12^. These observations point to the subtle differences in structure that can impact crystallization inhibitory behavior, which are not readily apparent from simple summing of chemical functional groups.

#### In vivo rat studies

ASDs were prepared with two cellulose derivatives, namely CPHPC-106 and CA Sub. These polymers were chosen based upon a consideration of their aqueous solubility and ability to inhibit enzalutamide crystallization. CA Sub was an extremely effective crystallization inhibitor, whereby crystallization was delayed for > 16 h, but has a low to moderate aqueous solubility of 3 mg/mL^10^. In contrast, CPHPC-106 delayed crystallization for only ~ 1 h, but has much higher aqueous solubility (43.5 mg/mL)^25^. Therefore, these polymers have contrasting properties in terms of crystallization inhibition and solubility. Hoy solubility parameters were calculated to qualitatively describe the relative hydrophilicity of the cellulose derivatives in their unionized state (Table 1). The polar and hydrogen bonding components of the solubility parameter were higher for CPHPC-106 than for CA Sub indicating that the former polymer is more hydrophilic, consistent with the aqueous solubility measurements. Enzalutamide is more hydrophobic than any of the polymers based on the total solubility parameter, as well as the calculated Log P, which was 4.75.

**Table 1:** Hoy solubility parameters of enzalutamide and select cellulose-based polymers.

Compound	Total solubility parameter (δ_t_) (J^1/2^ cm^−2/3^)	Polar solubility parameter (δ_p_) (J^1/2^ cm^−2/3^)	Hydrogen bonding solubility parameter (δ_h_) (J^1/2^ cm^−2/3^)	Dispersive solubility parameter (δ_d_) (J^1/2^ cm^−2/3^)	Aqueous solubility (mg/mL)
Enzalutamide	31.4	4.3	15.2	27.1	0.042^a^
HPMCAS-MF	25.8	16.0	14.6	14.1	23.4
CA Sub	21.0	12.1	8.2	15.1	3.0
CPHPC-106	23.4	14.4	11.4	14.4	43.5

^a^Amorphous solubility.

**Figure 2:**
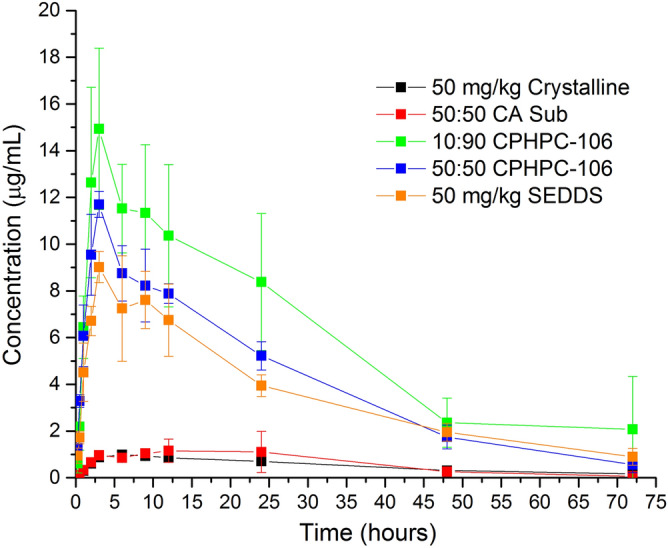
Plasma concentration versus time profiles for enzalutamide ASDs prepared with novel cellulose derivatives, SEDDS prepared with Labrasol, and a crystalline slurry. Formulations contained 4.5 (SEDDS formulation), 10 (CPHPC-106) or 50% CPHPC-106 and CA Sub) by weight of drug.

Most existing ASD polymers only work well at relatively low drug loadings, and as a result many ASDs consist of 90% polymer and 10% drug by weight. This is a significant drawback for formulation utility, especially for lower potency drugs; it can cause higher formulation costs, inconveniently large dosage form size, or necessitate taking multiple pills per dose. Therefore two different drug loadings, 10% and 50%, were tested for ASDs of enzalutamide with CPHPC-106 ASDs. Only one drug loading, 50%, was explored for enzalutamide/CA Sub ASDs due to low polymer availability. The in vivo performance of each of these ASDs was compared to two reference formulations, a crystalline suspension, and a formulation that mimics the commercial formulation (Fig. 2). The commercial formulation is a self-emulsifying drug delivery system (SEDDS) prepared with Labrasol, a non-ionic surfactant, and has a very low drug loading of only 4.5%. The absolute bioavailability, F, of the formulations and crystalline slurry was estimated from the following equation:

1 $$F = \frac{{AUC_{{0 - inf_{{formulation_{{50\frac{\text{mg}}{{\text{kg}}}dose}} }} }} }}{{5 \times AUC_{{0 - inf_{{IV_{{10\frac{\text{mg}}{{\text{kg}}}dose}} }} }} }}.$$

Equation (1) assumes a linear dose response following administration by intravenous bolus, as observed for for several structurally diverse compounds in rats^28–30^.

Oral administration of the ASDs to rats and subsequent pharmacokinetic analysis of enzalutamide plasma concentrations showed that the 10:90 Enz:CPHPC-106 ASD resulted in the highest area under the curve (AUC), and afforded the highest maximum plasma concentration (C_max_). The 50:50 Enz:CPHPC-106 ASD showed a very similar profile to the lipid-based formulation, in spite of the tenfold increase in drug loading, with both systems showing good bioavailability relative to the crystalline reference. In contrast, enzalutamide exhibited poor bioavailability from the CA Sub ASD, yielding an exposure profile similar to that of the crystalline slurry, although an extended absorption window was observed and the time until the maximum concentration (T_max_) was delayed relative to the crystalline reference (Table 2) leading to a longer absorption half-life (t_1/2abs_). Crystals were not observed for the CA Sub ASD in the aqueous suspension for up to 5 h based on evaluation with a polarized light microscope, therefore the low plasma concentrations cannot be due to crystallization of enzalutamide from the formulation. The trend of C_max_, F, and AUC was: 10:90 Enz:CPHPC-106 > 50:50 Enz:CPHPC-106 > SEDDS > 50:50 Enz:CA Sub = crystalline slurry (pharmacokinetic parameters summarized in Table 2). The crystalline slurry and 50:50 CA Sub ASD exhibited low bioavailability of approximately 4% whilst the SEDDS and CPHPC-106 ASDs all had approximately 9 × higher absolute bioavailability.

**Table 2:** Pharmacokinetic parameters following dosing of different enzalutamide formulations (50 mg/kg oral dose).

Formulation	AUC_0–inf_ (µg h/mL)	C_max_ (µg/mL)	T_max_ (h)	t_1/2_ (h)	t_1/2abs_ (h)	F^a^
Crystalline Slurry	43.1 (4.6)	1.04 (0.03)	5.0 (1.0)	22.2	1.9	3.6 (0.3)%
50:50 CA Sub	45 (14)	1.44 (0.34)	12.0 (6.2)	12.1	3.6	4.3 (2.4)%
SEDDS	283 (11)	9.34 (0.42)	4.0 (1.0)	20.7	1.1	31.5 (3.3)%
50:50 CPHPC-106	305 (19)	11.7 (0.3)	3.0 (0.0)	14.7	0.9	28.4 (2.2)%
10:90 CPHPC-106	516 (137)	15.3 (2.1)	2.7 (0.3)	15.1	1.0	46.0 (18.6)%

Values in parentheses are standard deviations, n = 3.

^a^F, absolute bioavailability, was estimated from intraveneous bolus dosed at 10 mg/kg AUC_0–inf_.

#### In vitro diffusion cell mass flowrate and comparison to in vivo AUC

**Figure 3:**
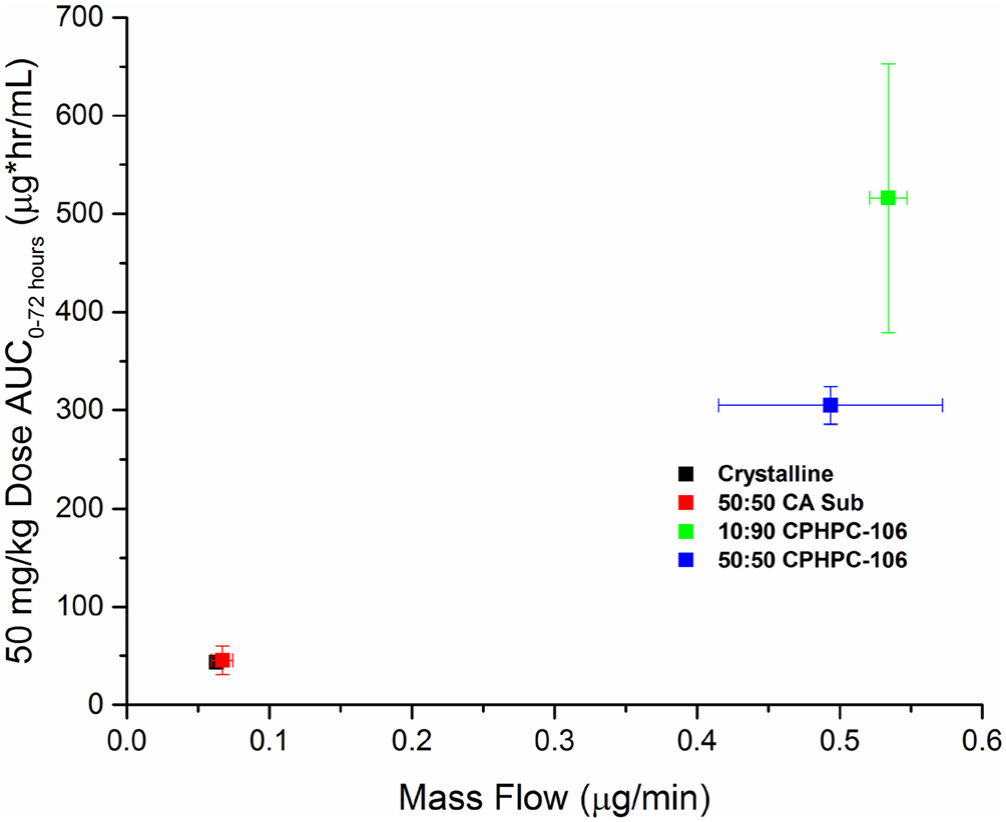
In vitro mass flow rates vs. in vivo AUC (0–72 h) of enzalutamide formulations. Mass flow rate was measured in a side-by-side diffusion cell after formulation equivalent to 100 μg/mL enzalutamide was added to the donor compartment. Rats were dosed with 50 mg/kg enzalutamide.

The mass flow rate of enzalutamide across an artificial membrane was investigated for all ASD formulations to compare the amount of free enzalutamide present in the suspensions dosed in the in vivo study and to determine whether this correlated with the amount of drug absorbed in vivo (Fig. 3). The highest in vitro mass flow rates were observed for solutions derived from dissolution of the 10:90 Enz:CPHPC-106 and 50:50 Enz:CPHPC-106 ASDs. The crystalline slurry control sample and 50:50 Enz:CA Sub ASD had the lowest mass flow rates, almost 5 times lower than that of 10:90 CPHPC-106 ASD. The samples with lower mass flow rates, as measured in the in vitro diffusion experiments, yielded low AUC values in the rat oral absorption studies, as summarized in Fig. 3.

## Discussion

Enzalutamide crystallizes rapidly from supersaturated solutions, thus, to formulate an effective ASD, the polymer must be an effective crystallization inhibitor, while also facilitating release of the drug from the dispersion. To enhance the dissolution rate of a hydrophobic drug such as enzalutamide, the polymer must be sufficiently hydrophilic to dissolve in a reasonable timeframe, enabling release of the drug into the medium. However, amphiphilic polymers have been found to be generally more effective crystallization inhibitors than very hydrophilic polymers^31,32^. Recent molecular dynamics simulations^11^ suggest that cellulose derivatives interact with high log P drugs in aqueous solution through the hydrophobic substituents on the cellulose backbone. However, polymers lacking sufficient hydrophilic groups tend to self-interact in water rather than forming drug-polymer interactions^11^, thus hydrophilic groups are also needed to interact with water and solvate the polymer. Therefore, the polymer must contain the “right balance” of hydrophobic and hydrophilic substituent groups to be an effective polymer for ASD formulations. Further, apparently small changes in chemical structure can lead to large differences in properties such as effectiveness as a crystallization inhibitor^11,33^.

We observe this exquisite balance between polymer properties that lead to effective amorphous solid dispersion in this study. First, the ability of structurally diverse polymers to delay enzalutamide crystallization from supersaturated solutions can be considered. One unresolved question is: over what timeframe must this inhibition persist to lead to improved bioavailability? Clearly, given that gastrointestinal transit occurs over several hours, it might be inferred that an extended inhibition duration (several hours) is required to improve absorption. However, for many drugs, the window of absorption is actually quite short, particularly if absorption only occurs in a specific region of the gastrointestinal tract. Out of the 24 polymers tested, 4 (HPMCAS, CA Sub, CAAd 3CES HE, and ECCP-B) inhibited crystallization for longer than 5 h, ample time to enable transit from the stomach to, and through, the small intestine. However, virtually all of the polymers showed some inhibitory effect. Since there were no obvious chemical or structural features that correlated with crystallization inhibition among the group of compounds tested, screening studies such as these are essential to identify leading polymer candidates. Intuitively, there is an expectation that polymers which inhibit crystallization for longer periods of time will be better ASD polymers; however, this ignores other important polymer characteristics discussed above. Hence, while screening for crystallization inhibition is important, it should clearly be combined with other in vitro tests to better identify new polymers suitable for ASD applications. Since the most appropriate in vitro tests are still under discussion, correlation to in vivo studies provides essential feedback on the development of appropriate surrogate tests.

Importantly, we note from the in vivo studies that a polymer which is not the most effective crystallization inhibitor, CPHPC-106, leads to substantially improved absorption relative to the extremely effective crystallization inhibitor, CA Sub, as shown by the 9 × higher bioavailability of CPHPC-106 ASD. This result highlights that there is indeed an interplay between effectiveness as a crystallization inhibitor and other key polymer properties, most notably polymer solubility in this instance. CPHPC-106 has a high aqueous solubility^25^ but delayed crystallization for only 1 h. Conversely, CA Sub inhibited crystallization for 16 h but has lower aqueous solubility^10^. CPHPC-106 contains oligo(hydroxypropyl) substituents, themselves somewhat hydrophilic, some of which are capped with a C5 side chain containing a terminal COOH group. CA Sub on the other hand lacks polar, neutral hydroxyalkyl groups, and its carboxyl group is at the end of a suberate ester substituent, in which the carboxyl is at the terminus of an octamethylene tether. Thus the lower water solubility of CA Sub is unsurprising. The degree of substitution (DS) values of the ionizable COOH group differ by a seemingly slight amount; 1.06 for CPHPC-106 vs. 0.9 for CA Sub. This difference in polymer solubility presumably accounts, in part, for the different levels of molecularly dissolved drug from the two ASDs. Dissolving polymer from the ASD can trigger drug dissolution, and higher polymer concentration in solution should provide better stabilization of dissolved drug (and drug nanodroplets) against recrystallization. Molecularly dissolved drug can be evaluated using flux measurements to evaluate the rate of mass transfer across a membrane. It is generally accepted that only free drug is available for membrane transport, and that flux is directly proportional to the free drug concentration. Recent studies have shown correlations between the flux of a given formulation in an in vitro side-by-side diffusion cell and in vivo outcomes^6,23^. Herein, the mass flow rate measurements (Fig. 3) suggest that the amount of free drug evolved from the CA Sub dispersion is similar to that obtained from dissolution of the crystalline form, correlating well with the in vivo data where the AUC values for these two systems are comparable. Since no drug crystallization was observed for the CA Sub ASD, the low free drug concentration can be attributed to the low polymer solubility combined with strong drug-polymer interactions in the matrix^21^. The low free drug concentration observed in the mass flow experiment was confirmed by conducting a release study, which showed that the maximum drug concentration attained for the CA Sub ASD was only ~ 3 μg/mL, which is very close to that obtained by dissolving crystalline drug (Supplementary Fig. S1). Interestingly, enzalutamide release from the CA Sub dispersion is slower relative to dissolution of the crystalline drug. Polymer aqueous solubility alone cannot account for the low extent of drug release since measured thermodynamic solubility of CA Sub would indicate that it can completely dissolve in the volume of solvent present in the flux experiment, thus it is likely that the presence of the drug strongly suppresses the polymer dissolution. In contrast, much higher flux values are seen for the two CPHPC-106 dispersions, and correspondingly, much higher AUC values are obtained (Table 2). Thus, the flux measurements on the various formulations appear to be a good in vitro surrogate for rank ordering in vivo performance.

The excellent in vivo performance of the dispersions formulated with CPHPC-106 warrants further discussion, given the relatively poorer performance of this polymer as a crystallization inhibitor in our screening experiments. First, it should be noted that the local environment in vivo after dosing is very different from our lab experiment, in particular in terms of hydrodynamics, and fluid composition, where many endogenous substances such as bile salts are present. Both hydrodynamic conditions and bile salts are known to influence crystallization kinetics^34,35^ and as a result, crystallization may well occur over a longer timeframe in vivo relative to in vitro lab experiments. Second, drug is passively absorbed following in vivo dosing, diminishing the amount of drug remaining in the intestinal compartment, relative to the closed compartment lab experiment. Thus, the rate of absorption is likely to impact crystallization rate and extent. Consequently, if absorption is faster or occurs on a time scale similar to that of crystallization, dosing formulations that give rise to supersaturated solutions, which lead to faster passive diffusion through the membrane, will increase the amount of drug reaching the systemic circulation for a drug with solubility limited absorption (assuming no complicating issues such as extensive first pass metabolism and/or efflux). It is of interest to note that the half-life for absorption (Table 2) of the CPHPC-106 dispersions is ~ 1 h. This confirms that a substantial portion of the drug is absorbed prior to crystallization. Further, the half-life is similar to that of the liquid SEDDS formulation (in which the drug is pre-dissolved) which suggests that dissolution is not the rate limiting step for the CPHPC-106 dispersions. In contrast, the absorption half-lives for the crystalline suspension and the CA Sub dispersion are 1.9 h and 3.6 h, respectively, which is consistent with lower luminal concentrations and slower dissolution rates. It should also be noted that the dispersion containing a 50% drug loading with CPHPC-106 has performance slightly better than that of the SEDDS formulation (which contains only 4.5% drug loading). Thus, this ASD formulation offers a significant potential advantage in terms of patient compliance with regard to final dosage form size and/or number of dosage units to be consumed, since considerably less of the CPHPC-106 excipient is required to achieve a formulation with comparable in vivo performance to the commercial formulation. For chronically ill patients taking multiple drugs, reducing the pill burden by decreasing the size or number of the dosage forms to be taken is of paramount importance.

## Conclusions

Several effective solution crystallization inhibitors of enzalutamide were identified from a cohort of cellulose derivatives newly synthesized as ASD polymer candidates. Amorphous solid dispersions were subsequently fabricated from one of the most effective crystallization inhibitors, and a more water soluble polymer that was less effective in in vitro crystallization inhibition studies. In vitro and in vivo tests carried out to characterize the new formulations showed a five-fold improvement in the extent of enzalutamide absorption from ASD with CPHPC-106, the polymer that was the less effective crystallization inhibitor, relative to a crystalline control. In contrast, a formulation with the polymer that was a more effective crystallization inhibitor, but which was less soluble, yielded minimal improvements in oral absorption relative to the crystalline control. In vitro flux experiments were a useful approach to rank order the various formulations in terms of the rate of membrane transport, which in turn showed a good correlation with the in vivo results. This study highlights the fact that overall performance of an ASD formulation is a complex interplay of drug and polymer properties, and provides further illumination to the nature of that interplay.

## Methods and materials

### Materials

 Abbreviations and details about the polymers used are summarized in Table 3. The following excipients were from commercial sources: HPMCAS-MF (Shin-Etsu Co. Ltd, Tokyo, Japan), company), PVPVA (BASF, Ludwigshaven, Germany), HPC (Ashland Inc., Covington, Kentucky), PVP K29/32 (BASF, Ludwigshaven, Germany), and Labrasol ALF (Gattefosse, Lyon, France). The novel cellulose derivatives were synthesized as described previously (abbreviations defined and compositions given in Table 1): CPHPC-106^25^, CPHPC-282^25^, CPHPC-069^25^, CPTod-202^11^, CAAd PEG^11^, CAAd HOEt^11^, CAAdP 0.85^36^, CAAd 0.67^36^, CA Sub^36^, CAAd 3CES HE^11^, CAUn 3CES TAE^11^, MC-Ad^24^, MCCP-A^24^, MCCP-B^24^, ECCP-A^24^, ECCP-B^24^, ECCP-C^24^, and ECCP-D^24^. Enzalutamide was obtained from ChemShuttle (Hayward, California). Cellulose acetate phthalate (CA Ph) was from Sigma-Aldrich (St. Louis, Missouri), and all organic solvents used were supplied by Fisher Scientific (Hampton, New Hampshire).

**Table 3:** Name, abbreviation, and structure of polymers used in this study.

Name	Abbreviation	Structure	Substituent/AHG
Hydroxypropyl methyl cellulose acetate succinate (MF grade)	HPMCAS		
Hydroxypropyl methyl cellulose (E3 grade)	HPMC		
Poly(vinylpyrrolidone) vinyl acetate VA 64	PVPVA		
Poly(vinylpyrrolidone) K 29/32	PVP		
Cellulose acetate phthalate	CA Ph		
Hydroxypropyl cellulose	HPC		
5-Carboxypent-1-yl hydroxypropyl cellulose^26^	CPHPC-106		DS(HP): 2.20 MS(HP): 4.40 DS(CP): 1.06
5-Carboxypent-1-yl hydroxypropyl cellulose^26^	CPHPC-282		DS(HP): 2.20 MS(HP): 4.40 DS(CP): 2.82
5-Carboxyprop-1-yl hydroxypropyl cellulose^26^	CPHPC-069		DS(HP): 2.2 MS(HP): 4.4 DS(CPr): 0.69
Cellulose backbone structure			
Cellulose propionate trioxodecanoate^11^	CPTod-202		DS(Pr): 0.98 DS(TOD): 2.02
Cellulose acetate adipate poly(ethylene glycol) ester^11^	CAAd PEG		DS(Ac): 1.82 DS(Ad): 0.56
Cellulose acetate adipate hydroxyethyl ester^11^	CAAd HOEt		DS(Ac): 1.82 DS(Ad): 0.56
Cellulose acetate adipate propionate^37^	CAAdP 0.85		DS(Ac): 0.04 DS(Pr): 2.09 DS(Ad): 0.85
Cellulose acetate adipate^37^	CAAd 0.67		DS(Ac): 1.82 DS(Ad): 0.67
Cellulose acetate suberate^37^	CA Sub		DS(Ac): 1.82 DS(Sub): 0.90
Cellulose acetate 3-(2-carboxyethylthio)-adipate, hydroxyethyl ester^11^	CAAd 3CES HE		DS(Ac): 1.82 DS(Ad): 0.79
Cellulose acetate 9-(2-carboxyethylthio)-Undecanoate, 2-(trimethylammonio)-ethyl ester^11^	CAUn 3CES TAE		DS(Ac): 1.82 DS(Un): 0.67
Methyl cellulose adipate^25^	MCAd		DS(Me): 1.62 DS(Ad):1.1
Methyl 5-carboxypentyl cellulose^25^^a^	MCCP-A		DS(Me): 1.62 DS(CP): 1.37
Methyl 5-carboxypentyl cellulose^25^^a^	MCCP-B		DS(Me): 1.62 DS(CP): 1.37
Ethyl cellulose backbone			
Ethyl 5-carboxypentyl cellulose^25^^a^	ECCP-A	Starting source commercial ethyl cellulose	DS(Et): 2.58 DS(CP): 0.38
Ethyl 5-carboxypentyl cellulose^25^^a^	ECCP-B	Starting source commercial ethyl cellulose	DS(Et): 2.58 DS(CP): 0.38
Ethyl 5-carboxypentyl cellulose^25^^b^	ECCP-C	Starting source wood pulp	DS(Et): 2.19 DS(CP): 0.36
Ethyl 5-carboxypentyl cellulose^25^^b^	ECCP-D	Starting source commercial methyl cellulose	DS(Et): 2.2 DS(CP): 0.56

Note that all structures above are not meant to imply regioselective substitution; they are depicted this way only for clarity and simplicity.

*AHG* anhydroglucose unit.

^a^Polymers differ only in how they are hydrogenated in the final synthetic step.

^b^Polymers differ in DS (degree of substitution, Et) of starting material and therefore product from those above.

#### Solubility parameter

Solubility parameters were calculated using Hoy’s method^37^. In short, group contributions to the polymer repeat unit are used to calculate the solubility parameter which can be divided into separate intermolecular interactions: hydrogen bonding, dispersive, and polar. The values for each molecular moiety molar attraction functions are summed and the value for the solubility parameter is calculated using the following equation:

2 $$\delta_{t} = \sqrt {\delta_{p}^{2} + \delta_{h}^{2} + \delta_{d}^{2} } ,$$

where the subscript t is for the total, p for polar, h for hydrogen bonding, and d for dispersive component.

#### Log P

The log P of enzalutamide was calculated using MarvinSketch 17.22.0 (ChemAxon Ltd, Hungary).

### Formulation preparation

#### Self-emulsifying drug delivery system (SEDDS)

SEDDS were prepared by dissolving crystalline enzalutamide in Labrasol (Gattefosse, Saint-Priest, France) with a drug loading of 4.5 wt%.

#### ASD preparation

ASDs of enzalutamide were prepared by dissolving enzalutamide and polymer in a common organic solvent, then rapidly removing the solvent by rotary evaporation. A Buchi Rotovapor—R (New Castle, Delaware) with a Yamato BM 200 (Tokyo, Japan) water bath maintained at 25 °C was used to prepare the ASDs, followed by additional drying under vacuum at 35 °C for 1 h to remove residual solvent. Samples were then ground to a powder using a mortar and pestle and stored in a desiccator prior to use.

Three different single-polymer ASDs were prepared: 50% drug loading with CA Sub, and 10% and 50% drug loadings with CPHPC-106. Methanol was used as common solvent to prepare CPHPC-106 ASDs and tetrahydrofuran was used as common solvent to prepare CA Sub ASDs.

### In vitro experiments

#### Nucleation induction time determination

The average time to detect the onset of crystallization for supersaturated solutions of enzalutamide was determined in phosphate buffer (pH 6.5, 50 mM) containing pre-dissolved polymer using an in situ UV dip probe as described by Mosquera-Giraldo et al.^11^. The nucleation induction time was defined as the time when the first signs of crystallization could be detected, and was determined as the point where there was an observed decrease in the absorbance maximum and a concurrent increase in the baseline signal (observed at a wavelength at which enzalutamide does not absorb, and therefore used as a measure of turbidity). For enzalutamide the absorbance maximum is 237 nm and the baseline wavelength used was 446 nm. Some polymers did not readily dissolve in the buffer, thus 1–10 ppm of polymer dissolved in an organic solvent was added to the solution to disperse/dissolve the polymer (see Supplementary Table S1). Next, a methanolic solution of enzalutamide (245 µL, 10 mg/mL) was added to the polymeric solution (35 mL) magnetically stirred at 300 rpm and maintained at 37 °C, leading to an initial enzalutamide concentration of 70 µg/mL. The experiments were performed in triplicate.

#### Mass flow-rate in side-by-side diffusion cell

The mass flow rates of enzalutamide formulations were measured using the method described in Wilson et al*.*^6^ In brief, ASDs were stirred for 1 h in phosphate buffer (30 mL, pH 6.5, 50 mM), at 37 °C, prior to transfer to the donor compartment of side-by-side diffusion cell to yield a concentration of 100 µg/mL enzalutamide (PermeGear, Hellertown, PA). The donor compartment was separated from the receiver compartment by a regenerated cellulose dialysis membrane with MW cutoff of 6–8 kDa (Spectra/Por 1, Spectrum Laboratories Inc., Rancho Dominguez, CA). The receiver compartment contained 30 mL of buffer. Samples (75 µL) were taken from the receiver compartment every 5 min and analyzed via the high performance liquid chromatography (HPLC) method described previously^6^. Compartments were maintained at 37 °C, and experiments were performed in triplicate.

### In vivo studies

All animal studies were approved by the AbbVie Institutional Animal Care and Use Committee (IACUC). Animals were housed in accordance with guidelines (Institute of Laboratory Animal Research. 2011. Guide for the care and use of laboratory animals. Washington (DC): National Academies Press) and regulations (Animal Welfare Regulations. 2009. 9 CFR §2.30–2.38, 3.1–3.19.) in an AAALAC accredited facility to ensure high standards of animal care and use.

#### Intravenous dose of enzalutamide

Studies were performed with male Sprague–Dawley rats (Charles River Laboratories, Wilmington, MA) to determine the in vivo pharmacokinetics of enzalutamide following intravenous dosing. The rats had free access to food and water throughout dosing. A solution in 10:90 dimethyl sulfoxide:polyethylene glycol-400 was prepared 1 h prior to dosing containing 10 mg/mL of enzalutamide and dosed at a volume of 1 mL/kg for a total dose of 10 mg/kg. Blood samples were obtained in K_2_EDTA coated tubes at the following time points after dosing: 6, 15 and 30 min; 1, 2, 3, 6, 9, and 12; and 1, 2, and 3 days. Plasma samples were centrifuged at 3000 *g* at − 4 °C for 10 min then stored at − 15 °C until analysis.

#### Orally dosed formulations

Studies were performed with male Sprague–Dawley rats (Charles River Laboratories, Wilmington, MA) to determine the in vivo systemic exposure and pharmacokinetics of enzalutamide from ASDs and SEDDS after oral dosing. The rats had access to food and water throughout dosing. A suspension of each ASD was prepared one hour prior to dosing, containing 5 mg/mL of enzalutamide, and dosed at 50 mg drug/kg animal weight. Blood samples were obtained in K_2_EDTA coated tubes at the following time points after dosing: 0.25, 0.5, 1, 2, 3, 6, 9, and 12 h; and 1, 2, and 3 days. Similarly, the SEDDS formulations were dosed at 50 mg/kg and samples were taken at the same time points. Plasma was prepared from blood samples by centrifugation at 3000 *g* at − 4 °C for 10 min, then stored at − 15 °C until analysis. Prior to analysis, the plasma samples were thawed and 10 µL was added to 275 µL acetonitrile with diclofenac internal standard. Samples were mixed and centrifuged with the supernatant being retained. The supernatant of each samples was diluted threefold with 0.1% formic acid in water. The samples were then analyzed on a Sciex API5500 mass spectrometer with a Turbo-Ion Spray source (*m/z* 465 > 209) (Framingham, Massachusetts) with a Fortis Pace C18 5 µm, 30 × 2.1 mm column (Fortis Inc., St. John’s, Canada) with a 0.1% formic acid in water and 0.1% formic acid in acetonitrile gradient. Analysis was performed with Sciex Analyst 1.6.2 software. The standard curves of enzalutamide had a least weighted appropriate regression fit up to 1/x^2^ quadratic and minimum R-squared value of 0.99. The plasma concentration data underwent non-compartmental curve fitting with WinNonlin (Certara, St. Louis, Missouri) to determine the area under curve from 0 to 48 h (AUC_0–48_) using the linear trapezoidal rule. The maximum plasma concentration, C_max_, was found directly from the plasma samples. Additional pharmacokinetic analysis was performed using PKSolver^38^, an add-in program for Microsoft Excel. Here, the data were fitted to a one compartment model assuming first order absorption and first order elimination.

## Supplementary information

Supplementary Information.
