# Supplementary material for: Amorphous solid dispersions of enzalutamide and novel polysaccharide derivatives: investigation of relationships between polymer structure and performance
Source: Sci Rep. 2020 Oct 28;10:18535. doi: 10.1038/s41598-020-75077-7 (PMC7595150; doi:10.1038/s41598-020-75077-7)
Supplement: Supplementary file 1 — Supplementary Information. [file 41598_2020_75077_MOESM1_ESM.docx]

**Supplemental Information**

**Amorphous Solid Dispersions of Enzalutamide and Novel Polysaccharide Derivatives: Investigation of Relationships between Polymer Structure and Performance**

Venecia R. Wilson, Xiaochun Lou, Donald J. Osterling, DeAnne F. Stolarik, Gary J. Jenkins, Brittany Nichols, Yifan Dong, Kevin J. Edgar, Geoff G. Z. Zhang, Lynne S. Taylor

Supplementary Figure S1. Comparison of the release rate of enzalutamide from the Ca Sub dispersion and the dissolution rate of crystalline enzalutamide at a drug dose of 100μg/mL.

Supplementary Table S1. Organic solvent used to pre-dissolve polymer prior to addition to pH 6.5 phosphate buffer.

| **Abbreviation** | **Organic Solvent** |
| --- | --- |
| HPMCAS | None |
| HPMC | None |
| PVPVA | None |
| PVP | None |
| CA Ph | None |
| HPC | None |
| CPHPC-106 | None |
| CPHPC-282 | THF |
| CPHPC-069 | THF |
| CPTod-202 | DMF |
| CAAd PEG | THF |
| CAAd HOEt | THF |
| CAAdP 0.85 | THF |
| CAAd 0.67 | THF |
| CA Sub | THF |
| CAAd 3CES HE | DMSO |
| CAUn 3CES TAE | DMSO |
| MC-Ad | DMF |
| MCCP-A | DMF |
| MCCP-B | DMF |
| ECCP-A | DMSO |
| ECCP-B | THF |
| ECCP-C | DMSO |
| ECCP-D | THF |
